# Effect of Seasonal Variation on Clinical Outcome in Patients with Chronic Conditions: Analysis of the Commonwealth Scientific and Industrial Research Organization (CSIRO) National Telehealth Trial

**DOI:** 10.2196/medinform.9680

**Published:** 2018-03-16

**Authors:** Ahmadreza Argha, Andrey Savkin, Siaw-Teng Liaw, Branko George Celler

**Affiliations:** ^1^ Biomedical Systems Research Laboratory University of New South Wales Kensington Australia; ^2^ Ingham Institute of Applied Medical Research School of Public Health and Community Medicine University of New South Wales Kensington Australia; ^3^ eHealth Research Program Commonwealth Scientific and Industrial Research Organisation Marsfield Australia

**Keywords:** telehealth, telemonitoring, seasonal variation, clinical trial, vital signs, chronic disease

## Abstract

**Background:**

Seasonal variation has an impact on the hospitalization rate of patients with a range of cardiovascular diseases, including myocardial infarction and angina. This paper presents findings on the influence of seasonal variation on the results of a recently completed national trial of home telemonitoring of patients with chronic conditions, carried out at five locations along the east coast of Australia.

**Objective:**

The aim is to evaluate the effect of the seasonal timing of hospital admission and length of stay on clinical outcome of a home telemonitoring trial involving patients (age: mean 72.2, SD 9.4 years) with chronic conditions (chronic obstructive pulmonary disease coronary artery disease, hypertensive diseases, congestive heart failure, diabetes, or asthma) and to explore methods of minimizing the influence of seasonal variations in the analysis of the effect of at-home telemonitoring on the number of hospital admissions and length of stay (LOS).

**Methods:**

Patients were selected from a hospital list of eligible patients living with a range of chronic conditions. Each test patient was case matched with at least one control patient. A total of 114 test patients and 173 control patients were available in this trial. However, of the 287 patients, we only considered patients who had one or more admissions in the years from 2010 to 2012. Three different groups were analyzed separately because of substantially different climates: (1) Queensland, (2) Australian Capital Territory and Victoria, and (3) Tasmania. Time series data were analyzed using linear regression for a period of 3 years before the intervention to obtain an average seasonal variation pattern. A novel method that can reduce the impact of seasonal variation on the rate of hospitalization and LOS was used in the analysis of the outcome variables of the at-home telemonitoring trial.

**Results:**

Test patients were monitored for a mean 481 (SD 77) days with 87% (53/61) of patients monitored for more than 12 months. Trends in seasonal variations were obtained from 3 years’ of hospitalization data before intervention for the Queensland, Tasmania, and Australian Capital Territory and Victoria subgroups, respectively. The maximum deviation from baseline trends for LOS was 101.7% (SD 42.2%), 60.6% (SD 36.4%), and 158.3% (SD 68.1%). However, by synchronizing outcomes to the start date of intervention, the impact of seasonal variations was minimized to a maximum of 9.5% (SD 7.7%), thus improving the accuracy of the clinical outcomes reported.

**Conclusions:**

Seasonal variations have a significant effect on the rate of hospital admission and LOS in patients with chronic conditions. However, the impact of seasonal variation on clinical outcomes (rate of admissions, number of hospital admissions, and LOS) of at-home telemonitoring can be attenuated by synchronizing the analysis of outcomes to the commencement dates for the telemonitoring of vital signs.

**Trial Registration:**

Australian New Zealand Clinical Trial Registry ACTRN12613000635763; https://www.anzctr.org.au/Trial/Registration/TrialReview.aspx?id=364030&isReview=true (Archived by WebCite at http://www.webcitation.org/ 6xLPv9QDb)

## Introduction

Telehealth systems in at-home, primary care, and hospital-based settings have been extensively investigated for more than 20 years [1-5]. Large health care organizations, such as the Veterans Administration in the United States and the National Health Service in the United Kingdom, have already adopted a range of telehealth solutions [6]. Employment of telehealth services for the management of patients with chronic conditions has progressively increased in recent years because of population aging and the increasing burden of chronic disease, along with the availability of low-cost monitoring technology.

Several trials have been carried out to analyze clinical, service, and economic benefits of telehealth systems [7,8]. These analyses are crucial to encourage wide-scale implementation of telehealth services. However, to the authors’ best knowledge, no study has examined the impact of seasonal timing of hospital admission and length of stay (LOS) on clinical outcomes of a home telemonitoring trial.

This paper discusses the possible effect of seasonal variations on the clinical outcomes of a recently completed Commonwealth Scientific and Industrial Research Organization (CSIRO) trial of home monitoring for chronic disease management, carried out at several locations along the east coast of Australia [9]. The aim of this trial was to investigate health care outcomes as well as clinical and economic benefits of telehealth systems by introducing a telehealth model of service based on at-home telemonitoring of vital signs to patients with a range of chronic conditions supervised in either in hospital-based or community-based settings. The clinical protocols for the trial [9], the data architecture design [10], decision support and statistical trend analysis of vital signs data [11], and the impact of telemonitoring on health care expenditure, hospital admissions, and LOS [8] have been published previously.

In this paper, we introduce a novel method to estimate seasonal trends in hospitalization data of 136 patients with cardiovascular disease, respiratory disease, and diabetes over 3 years (January 1, 2010 to December 31, 2012), recruited in the CSIRO National Telehealth Trial. The final hypothesis in this paper is to show that seasonal variations can be minimized to have little or no significant influence on the clinical outcomes reported for the CSIRO National Telehealth Trial.

## Methods

### Research Ethics Committee Approval

The CSIRO Human Research Ethics Committee (HREC) as well as five other local HRECs approved the clinical trial protocol for this study (Approval Number: 13/04, March 25, 2013).

### Patient Selection

In this trial, 1429 eligible patients from hospital lists provided by local health districts and patients known to clinical staff formed a Master Register. The local health districts were located in the states of Queensland, New South Wales, the Australian Capital Territory, and Tasmania. Inclusion criteria were thoroughly described in a previous article [9]; for convenience, we briefly summarize them here: age 50 years and older; at least two unplanned acute admissions during the previous 12 months or at least four unplanned acute admissions during the previous 5 years, with a principal diagnosis of coronary artery disease, congestive heart failure, hypertensive diseases, chronic obstructive pulmonary disease (COPD), asthma, or diabetes. Patients with compromised cognitive function, a neuromuscular disease, cancer, or a psychiatric condition were excluded from the trial. From the Master Register, 479 were deemed eligible and were contactable following individual screening.

**Figure 1:**
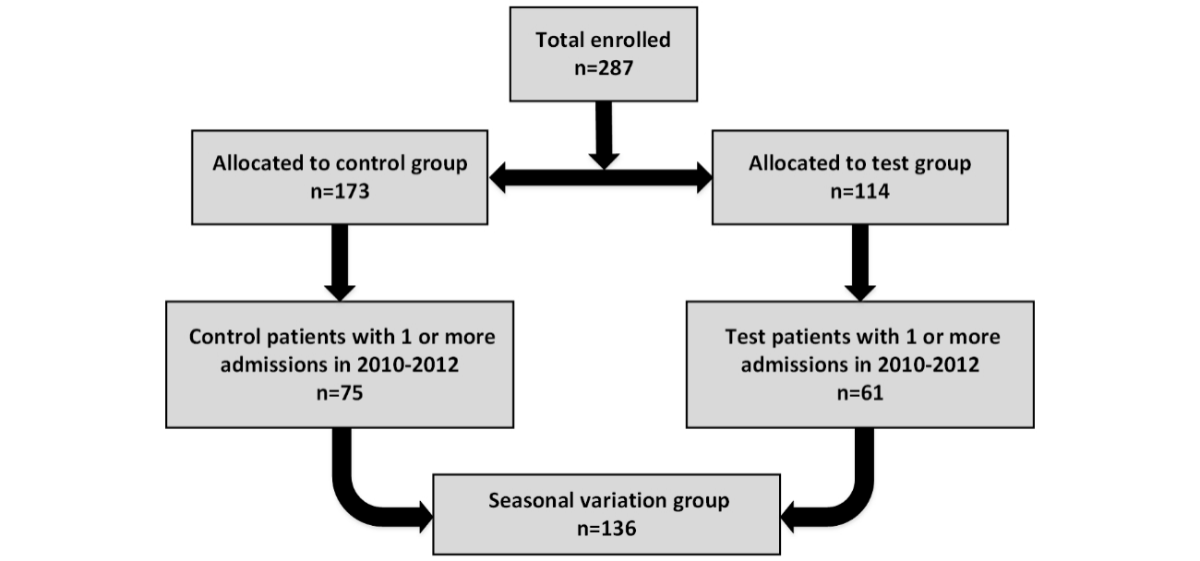
Final cohort of seasonal variation group.

Of the 479 eligible patients, only 287 could commence the trial, of which 114 were allocated to the telemonitoring test group and 173 were allocated to the control group [8]. The test patients were supplied with a telemonitoring system and trained on its use at installation, whereas the control group received only normal care through their primary care physician. Of the 287 patients monitored in this trial, we only considered patients who had one or more admissions in the years 2010 to 2012. With this additional inclusion criterion, 61 patients from the test group and 75 patients from the control group were selected to estimate the seasonal variation trends in the three years of 2010 to 2012. Figure 1 summarizes the patient selection process for this study.

However, because the telemonitoring intervention was only experienced by the 61 test patients, only these patients could be considered when evaluating the effect of seasonal variations on outcome variables, noting that the start of the intervention was synchronized for all test patients, considering each of the three climate subgroups separately.

### Definition of Seasons

The temperate zone along the eastern seaboard of Australia occupies the coastal hinterland of New South Wales, much of Victoria, and Tasmania. Hence, the four test sites in this trial are located in the temperate zone where seasons, in terms of European seasons applied to the southern hemisphere, are described as follows: summer (December to February), autumn (March to May), winter (June to August), and spring (September to November).

However, the fifth trial site, Townsville in Queensland, is within a subtropical zone, which is dominated by two distinct seasons: the wet season in summer (November to April) and the dry season in winter (May to October). Summer months in this city are generally hot and humid with day temperatures often around 29°C to 31°C and night temperatures around 20°C to 24°C. Winter months are generally warm to mild with day temperatures often around 25°C to 29°C and night temperatures around 13°C to 18°C.

### Regression Modeling

The number of hospital admissions and LOS were analyzed as outcome variables in this study. As discussed in a previous article [8], all the outcome variables of this trial, including admission rates and LOS, were expected to increase over time because the patients involved in this trial were chronically ill and aging. To remove the aging effects from the 3 years’ of hospitalization data, we fitted a linear model with the calendar months of the year as variables (3 years=36 months).

To implement this, the admission rate and LOS were summed for all patients within each calendar month of the year. The monthly time course of 3 years’ of data was then modeled using linear regression to identify statistically significant differences in admission rates and LOS slopes.

We used the “fit” command in the MATLAB (The MathWorks Inc) statistics toolbox to carry out linear regression. To obtain 95% prediction intervals, the command “predObs” was used to plot 95% prediction intervals. A 95% prediction interval is an estimate of an interval in which future observations will fall, with 95% probability, given what has already been observed.

Moreover, different standard goodness-of-fit measures, including the coefficient of determination (*R*^2^), the *R*^2^ value adjusted for degrees of freedom, the residual sum of squares, and the standard error or root mean square error were considered.

Following the derivation of a linear model (baseline) in this study, we replaced the absolute vales of outcome variables (rate of admission, number of hospital admissions, and LOS) with percentage deviation from the baseline. The deviation from baseline was defined as the distance between each observations point (outcome variable at each month) and its corresponding point at baseline, which was estimated by the linear regression line of best fit *.* Then, a yearly seasonal variation trend was obtained by averaging over each calendar month the percentage deviation from baseline over the 3 years’ of data available.

### Seasonal Effects on Outcome Variables

During interventions, the 61 test patients were monitored for a mean 481 (SD 77) days with no significant difference between average monitoring durations for female patients (mean 498, SD 82 days) and male patients (mean 463, SD 67 days). Of the 61 test patients, 87% (53/61) were monitored for periods exceeding 12 months.

As proposed in a previous article [8], a possible method to minimize the impact of seasonal variation on the outcomes of the telemonitoring trial is to synchronize medical, pharmaceutical, and hospital data to the date when the telemonitoring commenced, thus effectively smoothing the effect of seasonal variations. However, no accurate analysis was given in that article on the effect of time synchronization compared to using actual monitoring durations.

Hence, to compare these two methods, we compensated for the effect of seasonal variation on each patient monthly data point by using the yearly seasonal variation model obtained previously from 3 years’ of hospital data.

### Statistical Analysis

To determine the statistical significance of the differences between subgroups, a two-sample *t* test was performed for continuous variables and the Wilcoxon rank sum test was carried out for skewed variables. For describing baseline characteristics, we used means and standard deviations for continuous symmetrical variables and medians and 95% confidence intervals for skewed data.

Categorical variables are also presented as counts and percentages. All statistical tests were two-tailed, and *P*<.05 was considered statistically significant. Statistical analyses were performed using MATLAB (R2016b) and Microsoft Excel.

## Results

### Findings

Basic demographics of seasonal variation group in the study are given in Table 1. There were no significant differences in age between test patients in each of the five sites and between male and female patients. Test patients continued to be monitored in their own home, and no patient requested a relocation of their telemonitoring equipment to another location during the trial.

In this study, 58.1% (79/136) of the patients were male and 41.9% (57/136) were female. Most patients included in this study had more than one condition listed as a primary diagnosis, but for simplicity, primary disease conditions were grouped in the broad categories of cardiovascular disease (n=55), respiratory disease (n=66), and diabetes (n=15).

The 136 combined test and control patients were admitted to hospital 817 times during 2010 to 2012, with a total LOS of 3627 days. Adopting the same definition of season for the site in Queensland, the highest number of patients were admitted during spring (218/817, 26.7%) followed by winter (216/817, 26.4%), autumn (207/817, 25.3%), and summer (176/817, 21.5%). Excluding winter, the number of hospital admissions was significantly higher in spring compared to other seasons (*P* values versus winter, autumn, and summer were .86, .04, and .02, respectively).

However, LOS was higher during winter (1007/3627, 27.76%) followed by spring (990/3627, 27.30%), and autumn (916/3627, 25.26%). Overall LOS was shorter during summer (714/3627, 19.69%). Except for spring, LOS was significantly longer in winter compared to other seasons (*P* values versus spring, autumn, and summer were .62, .03, and .01, respectively).

We considered three different groupings because of substantially different climates: (1) Queensland (subtropical), (2) Australian Capital Territory and Victoria (temperate), and (3) Tasmania (colder). Regarding Queensland, because only two distinct seasons (winter and summer) are notable, we compared the variables for these two seasons. The number of hospital admission and overall LOS were significantly higher and longer in winter (hospital admissions=157, LOS=633 days) compared to summer (hospital admissions=98, *P*=.01; LOS=338 days, *P*=.02).

**Table 1:** Basic demographics of seasonal variation patients.

Location		Demographics					
		Patients, n	Patient age, mean (SD)	Male, n	Male patient age, mean (SD)	Female, n	Female patient age, mean (SD)
**Tasmania**							
	Test	23	70.3 (9.4)	14	70.5 (11.0)	9	69.9 (6.6)
	Control	45	72.7 (9.0)	25	73.5 (8.1)	20	71.6 (10.1)
**Australian Capital Territory**							
	Test	11	71.1 (7.9)	8	70.4 (8.4)	3	73.1 (7.4)
	Control	13	75.8 (7.8)	7	73.3 (7.4)	6	77.3 (8.4)
**Victoria**							
	Test	6	65.4 (6.1)	3	68.6 (4.5)	3	62.1 (6.4)
	Control	1	76.1 (0)	0	—	1	76.1 (0)
**Queensland**							
	Test	21	70.9 (10.5)	12	68.7 (8.9)	9	73.8 (12.4)
	Control	16	74.3 (7.5)	10	74.5 (7.9)	6	73.9 (7.6)
**Total**							
	Test	61	70.2 (9.2)	37	69.7 (9.1)	24	70.8 (9.5)
	Control	75	73.5 (8.4)	42	73.7 (7.7)	33	73.2 (9.3)
	All	136	72.0 (8.9)	79	71.9 (8.6)	57	72.2 (9.4)

For Australian Capital Territory and Victoria, the maximum number of admissions was in spring (43/119, 36.1%) followed by winter (34/119, 28.6%), autumn (21/119, 17.7%), and summer (21/119, 17.7%). Although hospital admissions were not significantly higher in spring compared to winter (*P*=.48), they were significantly higher than for autumn (*P*=.009) and summer (*P*=.01). Furthermore, the longest LOS was during spring (192/478, 40.2%), followed by winter (138/478, 28.9%), summer (75/478, 15.5%), and autumn (73/478, 15.1%). LOS was significantly longer in spring compared to autumn (*P*=.004) and summer (*P*=.01), but not significantly different from winter (*P*=.32), thus matching the results obtained for the number of hospital admissions. No significant differences were observed for Tasmania between seasons (hospital admissions: 107, 109, 114, 113; LOS: 522, 554, 645, 457 for winter, spring, autumn, and summer, respectively). These results are summarized in Table 2.

### Obtaining Seasonal Trends

**Figure 2:**
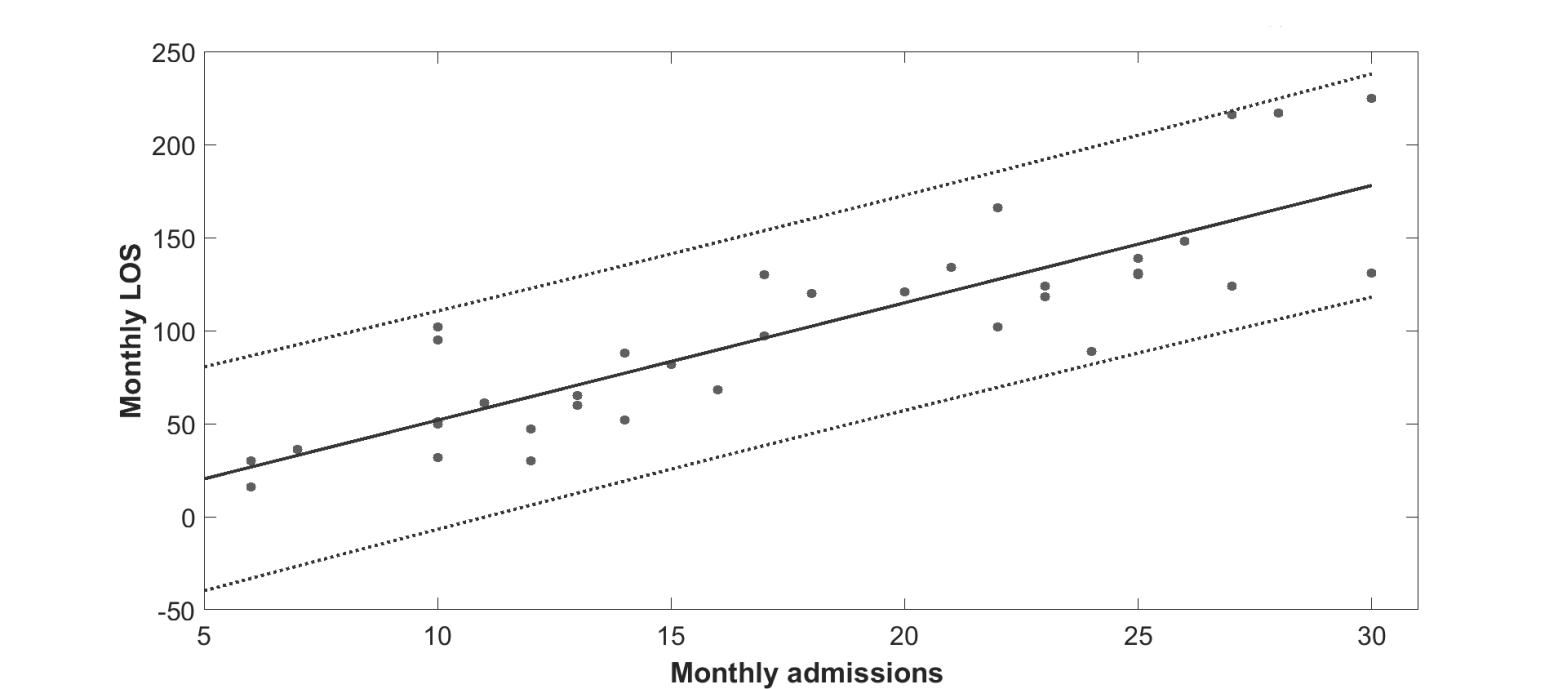
Length of stay (LOS) versus hospital admissions for 136 (test and control) patients in years 2010 to 2012. Correlation coefficient=0.86. Solid line is the linear regression line (slope=6.31, intercept=–11.17, R2=.73), and dotted lines are 95% prediction bounds (slope=4.98, 7.63 and intercept=–36.59, 14.24).

The objective here is to explain the procedure for obtaining seasonal trends. As mentioned earlier, three different climate subgroups were considered: (1) Queensland (subtropical), (2) Australian Capital Territory and Victoria (temperate), and (3) Tasmania (colder). As a result, three subtrends were obtained for the three climate subgroups, accordingly. Note also that due to space constraints, we only show the analysis for one subgroup (Queensland) here. Other seasonal trends for other subgroups were obtained using the same method. Additionally, because there was a strong positive correlation between number of hospital admissions and LOS (Figure 2), we only studied LOS as the outcome variable. The data in Figure 2 suggest that, for these patients, each admission resulted in an average LOS of 6.31 days.

**Table 2:** Seasonal variation in hospital admissions and length of stay (LOS).

Location		Season, n (%)				Total, n (%)	*P* (winter vs)^a^		
		Summer	Autumn	Winter	Spring		Summer	Autumn	Spring
**Tasmania**									
	Admissions	113 (25.5)	114 (25.7)	107 (24.2)	109 (24.6)	443 (54.2)	.51	.38	.74
	LOS	457 (21.0)	645 (29.6)	522 (24.0)	554 (25.4)	2178 (60.0)	.38	.62	.59
**Australian Capital Territory and Victoria**										
	Admissions	21 (17.7)	21 (17.7)	34 (28.6)	43 (36.1)	119 (14.6)	.08	.06	.48
	LOS	75 (15.7)	73 (15.3)	138 (28.9)	192 (40.2)	478 (13.2)	.14	.04	.32
**Queensland**									
	Admissions	98 (38.4)	—	157 (61.6)	—	255 (31.2)	.01	—	—
	LOS	338 (34.8)	—	633 (65.2)	—	971 (26.8)	.02	—	—

^a^*P* values were calculated using the Wilcoxon rank sum test.

### Removing Aging Effects Via Linear Regression Analysis

**Figure 3:**
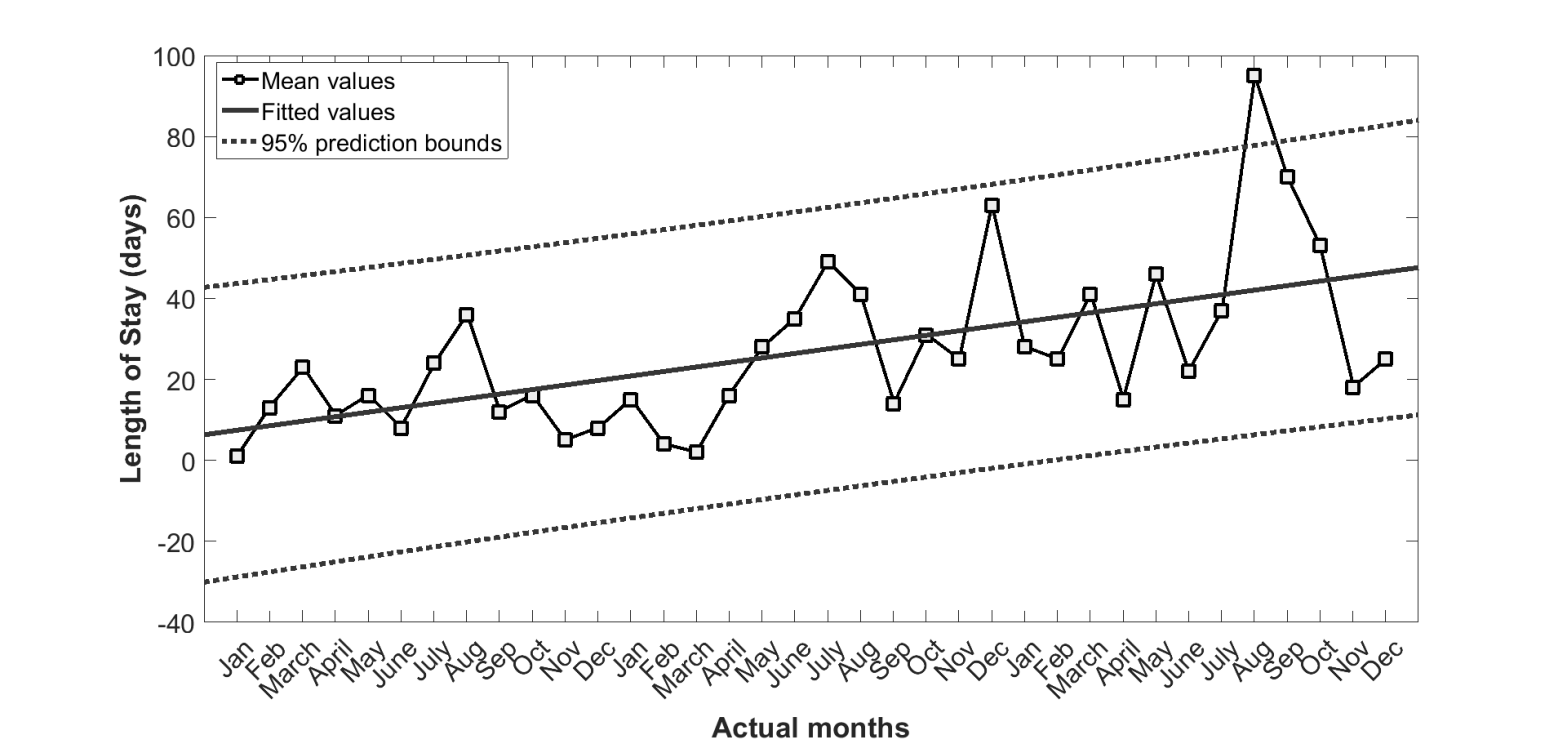
Length of stay for 37 (test and control) patients of Queensland subgroup in years 2010 to 2012. Solid line is the linear regression line (slope=1.12, intercept=6.32, R2=.33), and dotted lines are 95% prediction bounds (slope=0.56, 1.67 and intercept=–5.41, 18.06).

Figure 3 shows the LOS summed over each calendar month of the 3 years before the intervention for the Queensland subgroup. To estimate and remove the aging effects from the 3 years’ of hospitalization data, a linear model including the calendar months of the year as variables (3 years=36 months) was fitted. The solid line in Figure 3 is the linear regression line (LOS: slope=3.93, intercept=28.06 with *R*^2^=.60).

### Percentage of Deviation From Baseline

**Figure 4:**
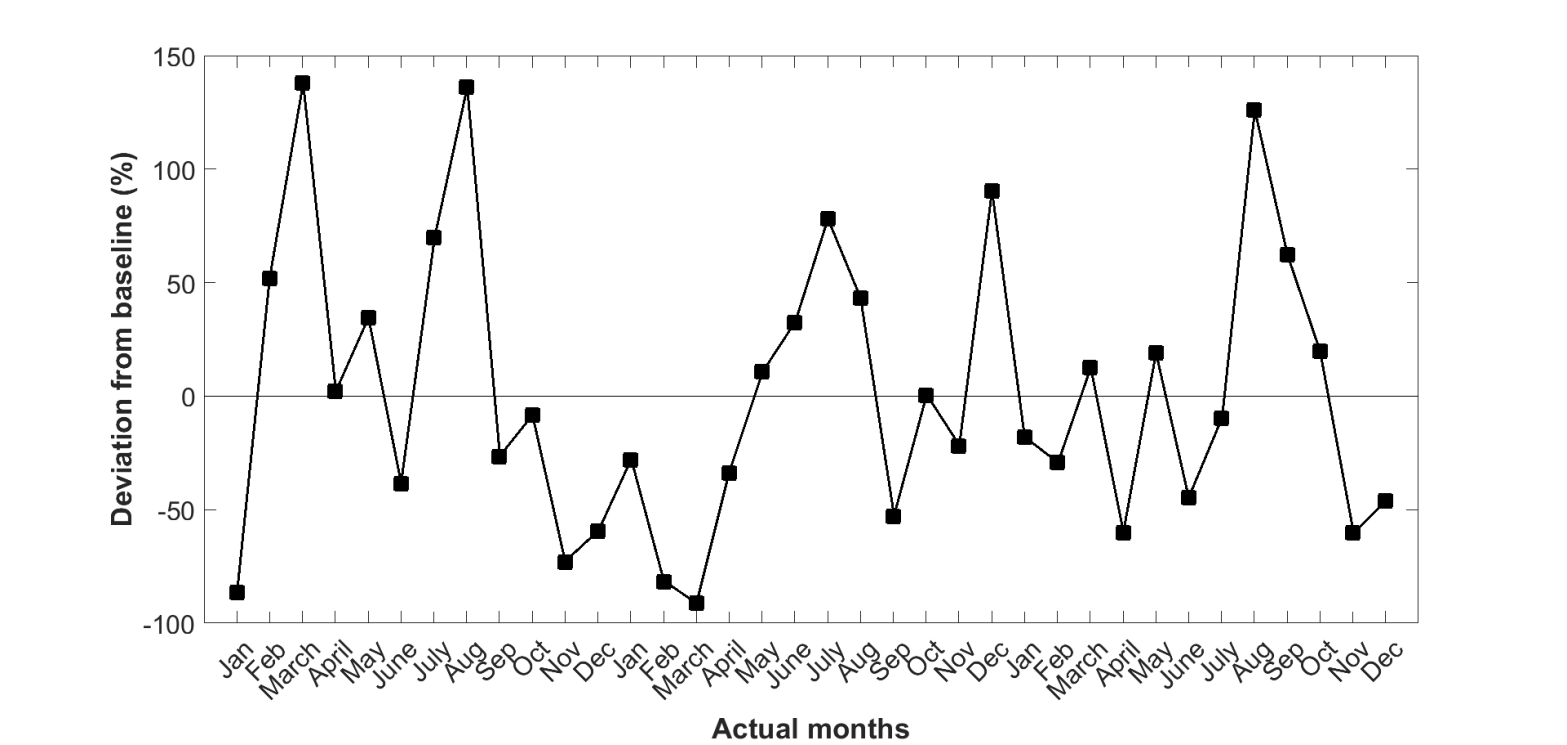
Deviation from baseline (fitted values) in length of stay of 37 (test and control) patients of Queensland subgroup in years 2010 to 2012.

**Figure 5:**
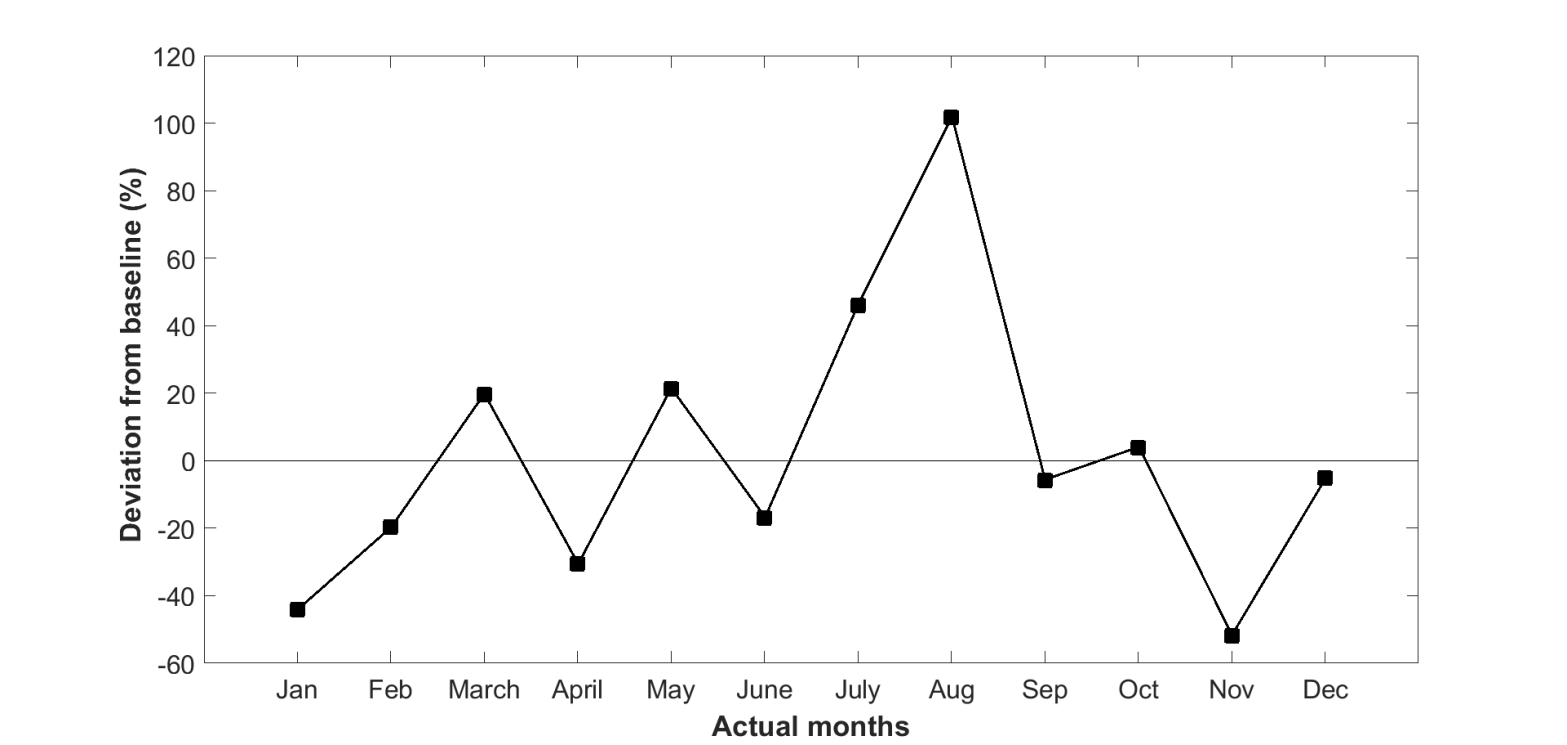
Average deviation from baseline: seasonal variation trend of length of stay in hospital for Queensland patients.

We then replaced the absolute values of LOS with the percentage deviation from the baseline trend line as shown in Figure 4. The seasonal variation trend (Figure 5) for LOS was then derived by averaging the 3 years’ of values in Figure 4.

### Distribution of Commencement Dates

**Figure 6:**
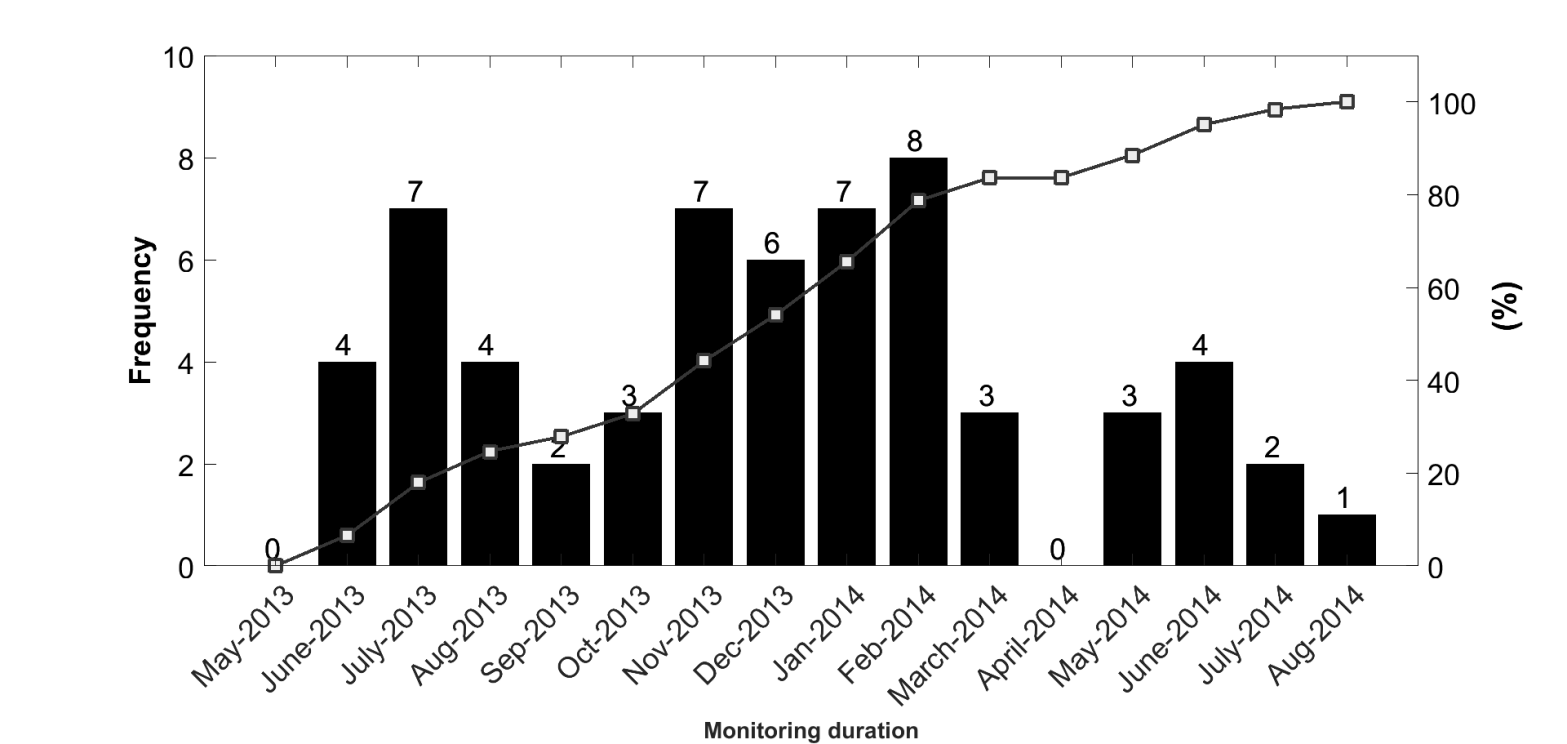
Distribution of commencement dates for monitoring of vital signs.

Figure 6 shows the wide distribution of commencement dates for test patients supplied with a device for the daily monitoring their vital signs.

### Influence of Synchronization of Commencement Days on Seasonal Variation

**Figure 7:**
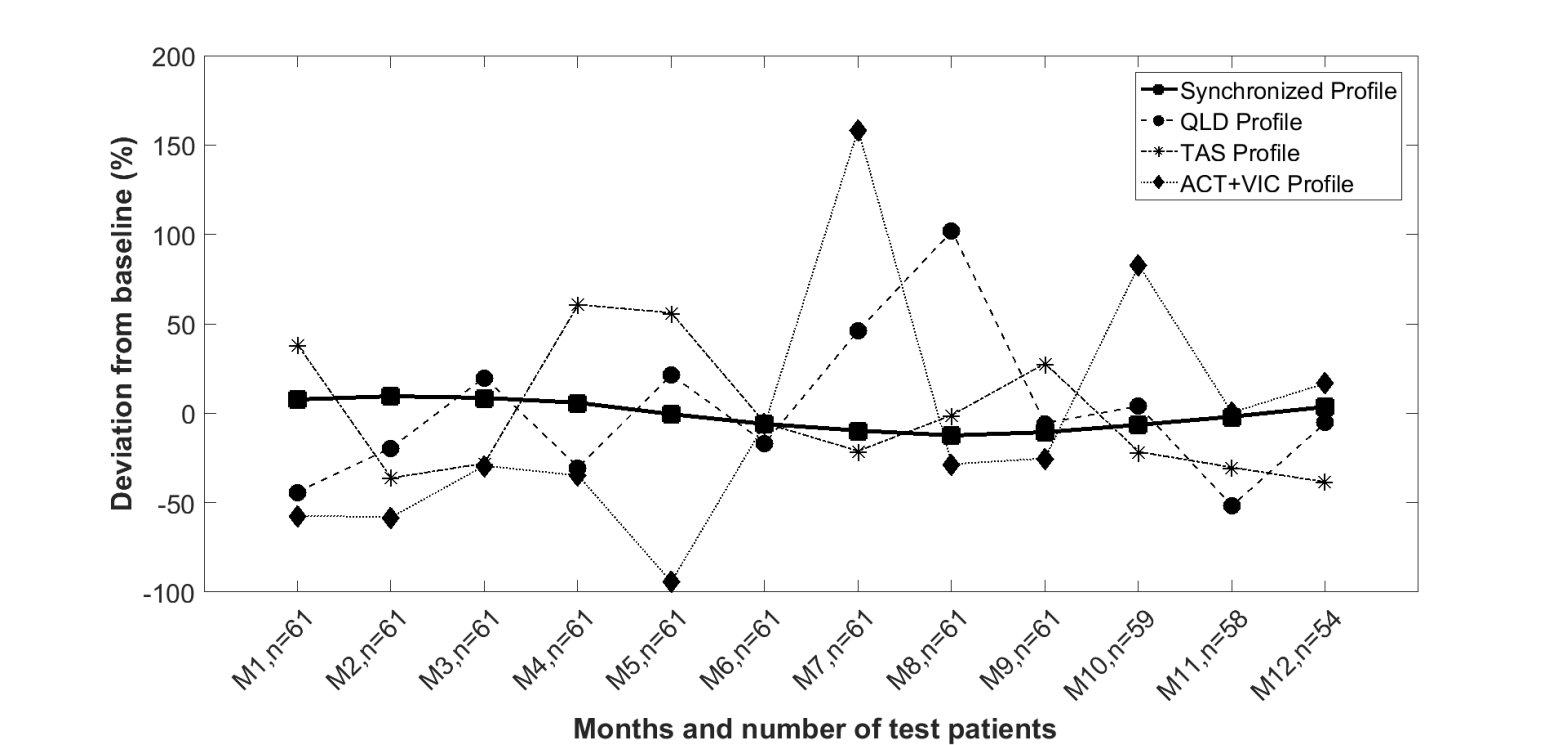
Estimated seasonal variation impact on length of stay with synchronized commencement days at different trial sites in Australia. QLD: Queensland, TAS: Tasmania, ACT: Australian Capital Territory, VIC: Victoria.

Seasonal annual variation for LOS is shown in Figure 7, calculated from the average trend of the previous 3 years prior to the start of intervention, for the three subgroups (Queensland, Tasmania, and Australian Capital Territory and Victoria). The seasonal variation in LOS shows that the maximum deviation from baseline was 101.7% (SD 42.2%), 60.6% (SD 36.4%), and 158.3% (SD 68.1%) for the Queensland, Tasmania, and Australian Capital Territory and Victoria subgroups, respectively.

**Figure 8:**
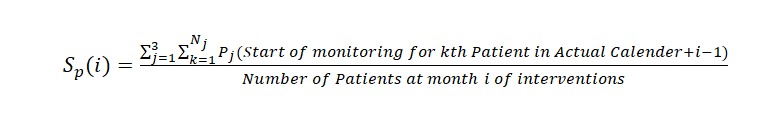
The formula to obtain the synchronized seasonal profile.

The synchronized profile shown in Figure 7 was derived by averaging the LOS calculated for the actual calendar month when monitoring began for each of the subsequent 12 calendar months. Note that month 1 after the start of monitoring for one patient could be March, whereas it could be September for another, as shown in Figure 6. Thus, for example, the value of the synchronized profile in the *i* th (*i*=1,...,12) month results from the ratio of the sum of the corresponding values of the obtained seasonal profiles at the first month of monitoring (ie, March or September) for all patients, and the number of patients monitored at the *i* th month. Similarly, this value can be calculated for subsequent months. In summary, the synchronized profile can be obtained by the formula in Figure 8.

In Figure 8, *S*_p_(*i*) denotes synchronized profile at *i* th month, *N*_j_ denotes the number of patients in *j* th (*j*=1,2,3) subgroups (ie, Tasmania, Queensland, and Australian Capital Territory and Victoria), and *P*_j_ is the seasonal profile achieved for *j* th subgroups.

As evident from Figure 7, by synchronizing the data to the start of monitoring, the impact of seasonal variation in LOS is greatly reduced to a peak of 9.5% (SD 7.7%), thus minimizing the impact of seasonal variations on the time course of LOS and other output variables.

Let us assume that the recruitment distribution is a Poisson distribution with lambda as the rate (mean) parameter (ie, the average number of patients recruited in a month). To identify lambda from the actual distribution of recruitments in the trial, we used the “poissfit” command of MATLAB, which returns the maximum likelihood estimate of the Poisson distribution, with lambda given by the data. The estimated value of lambda was 6.5.

 Using the lambda estimate, we created 100 sets of random numbers following the Poisson distribution by using *poissrnd(6.5,1,61)*, where 61 was the number of test subjects, and the achieved average maximum deviation from baseline, after synchronizing the analysis of LOS to the commencement of the intervention, was 9.0% (SD 7.5%). These values are quite close to the ones derived when the actual recruitment distribution was used, showing that the actual recruitment distribution is very close to a Poisson distribution.

Let us now assume that the recruitment distribution is a discrete uniform distribution (ie, the recruitment of patients is equally likely to occur during the whole duration of the intervention). Again, we created 100 sets of random numbers following a discrete uniform distribution by using *unidrnd(15,1,61)*, where 15 here is the total length of intervention in month.

 Synchronizing the analysis of LOS to the commencement of the intervention, the average maximum deviation from baseline was obtained as 4.9% (SD 3.0%), which is significantly smaller than the one obtained from the previous Poisson distribution. In other words, the impact of seasonal variation on the outcome variables of a telemonitoring trial can be minimized by evenly distributing recruitment over the entire monitoring duration and synchronizing the analysis of outcome variables to the commencement of the intervention.

## Discussion

The existence of seasonal variation in incidence of stroke, blood pressure, sudden death, myocardial ischemia, acute myocardial infarction, pulmonary embolism, lung function, and symptoms in COPD has been widely documented [12-17]. Seasonal variation also has an impact on the hospitalization rate of patients with a range of cardiovascular diseases [18,19], as well as acute myocardial infarction and angina in a western Sicily (Italy) hospital [20-21]. From these references, most admissions occur in the winter season for patients with cardiovascular disease from increased hypertension [22], ischemia [23,24], and recurrent infections [25].

The relationship between COPD exacerbation and seasonality has also been investigated in the Towards a Revolution in COPD Health [26] and Prevention of Exacerbations with Tiotropium in COPD [27] trials, both large international studies with more than 13,000 patients. These studies showed an increase in COPD exacerbations as well as an increase in hospitalization rate during the winter months. However, no association was observed in the tropics. This could be due to the fact that respiratory viruses are more prevalent in the cold months of temperate countries [28].

In the CSIRO National Telehealth Trial, a winter-spring predominance was evident in the seasonal variation in hospitalization and LOS both in the overall patient cohort as well as the Queensland and Australian Capital Territory and Victoria subgroups. This finding is partially in-line with the results of several other studies [12-17] performed in different countries with COPD and congestive heart failure patients showing a peak in winter.

The average LOS per admission was in summer (4.06 days) followed by autumn (4.42 days), spring (4.54 days), and winter (4.66 days). This reveals that an increase in the hospitalization rate coincided with a longer average LOS in cold months.

In Launceston, Tasmania, admissions were below average in spring and summer, increased rapidly in autumn (which coincided with high rainfall periods), and then dropped off again in winter before increasing again quite rapidly as winter ended.

The LOS broadly matches this pattern of admissions except for unexpectedly high LOS and below average rates of admissions in January. This anomaly is explained by local circumstances at Launceston base hospital, where new and inexperienced medical staff arrive in January to replace more experienced clinicians on leave, and have a tendency to keep patients in hospital longer as a precautionary measure.

Townsville in Queensland is a subtropical area with a rainy season in January, February, and March. Admissions were below average during the wet season, but increased rapidly following the end of the wet season, possibly due to increased pollen counts. However, LOS remained fairly static until August, at the end of winter, when they almost doubled. This is difficult to explain because peak average temperatures in winter are around 25°C in Townsville versus 12°C in Tasmania.

This study confirms the existence of a significant seasonal variation in hospital admissions as well as LOS in a recently completed CSIRO national trial of home telemonitoring of patients with chronic conditions, carried out at five locations along the east coast of Australia. Climactic and environmental conditions can also change year by year as shown in Figures 3 and 4, making the analysis of seasonal impacts more difficult to interpret.

We have shown that by synchronizing analysis of outcomes to the start of the intervention, the effect of seasonal variation on clinical outcome of an at-home telemonitoring trial can be significantly attenuated. To the authors’ knowledge, this is the first study to introduce a method to attenuate the effect of seasonal variations on the time course of outcome variables by synchronizing the analysis to the start of intervention for each patient.

This method recognizes that difficulties of recruitment of test patients in clinical trials are common, and patients may often be recruited over many months. This study suggests that by evenly distributing recruitment over the intervention duration and synchronizing the analysis of outcome variables to the commencement of the intervention, the confounding impact of seasonal variations can be minimized.

## Abbreviations

COPD: chronic obstructive pulmonary disease
CSIRO: Commonwealth Scientific and Industrial Research Organization
HREC: Human Research Ethics Committee
LOS: length of stay
